# Redistribution of Free- and Cell-Surface-Bound DNA in Blood of Benign and Malignant Prostate Tumor Patients

**Published:** 2015

**Authors:** O. E. Bryzgunova, S. N. Tamkovich, A. V. Cherepanova, S. V. Yarmoshchuk, V. I. Permyakova, O. Y. Anykeeva, P. P. Laktionov

**Affiliations:** Institute of Chemical Biology and Fundamental Medicine, Siberian Branch of the Russian Academy of Sciences, Prosp. Lavrentieva, 8, 630090, Novosibirsk, Russia; Novosibirsk State University, Pirogova Str., 2, 630090, Novosibirsk, Russia; Meshalkin Novosibirsk State Research Institute of Circulation Pathology, Rechkunovskaya Str., 15, 630055, Novosibirsk, Russia

**Keywords:** plasma, circulating DNA, DNase activity, prostate cancer

## Abstract

A direct correlation between the concentration of cell-free and cell-surface-bound circulating DNA (cfDNA and csbDNA, respectively) was demonstrated. Based on an inverse correlation between blood plasma DNase activity and the cfDNA concentration, blood DNases are supposed to regulate the cfDNA concentration. However, no correlation was found between the DNase activity in blood plasma and the csbDNA concentration, indicating that blood DNases are not involved in csbDNA dissociation from the cell surface. The possibility of DNA redistribution between cfDNA and csbDNA indicates that the total pool of circulating DNA (cfDNA + csbDNA) should be used for a correct analysis of marker DNA concentrations and data standardization.

## INTRODUCTION

The blood of healthy donors and cancer patients is known to contain constantly circulating extracellular DNAs. These circulating DNAs are found both in apoptotic bodies, nucleosomes, and the macromolecular protein complexes of plasma [1, 2] and on the surface of blood cells [1]. Previously, we demonstrated that the development of oncologic diseases such as breast cancer [3, 4] and lung cancer [5] is accompanied by an increase in the cfDNA concentration and a decrease in the csbDNA concentration. The relationships between these pools of circulating DNAs were not studied.

This article presents a study of the distribution of circulating DNAs between plasma and the surface of blood cells in prostate cancers.

## EXPERIMENTAL

Blood samples of healthy males (*n *= 40) at the age of 37–71 (47.4 ± 1.3) years, containing the prostate-specific antigen (PSA) at the concentration corresponding to the clinical norm (not exceeding 2.8 ng/mL), were received from the Central Clinical Hospital of the Siberian Branch of the Russian Academy of Sciences. Blood samples of newly admitted patients with prostate cancers at the age of 45–84 (70.2 ± 1.4) years were received from Municipal Clinical Hospital No.1; the PSA concentrations in a group of patients with benign hyperplasia (*n *= 25) and prostate cancer (*n *= 16) were 0–31.7 and 0–103.1 ng/mL, respectively (increased in 36 and 81% of cases). The study was conducted in compliance with the principles of voluntarism and confidentiality, according to the Fundamentals of Health Protection of Citizens in the Russian Federation. The disease stage was determined according to the TNM classification.

Collection and processing of blood and extraction of cfDNA from plasma, csbDNA from a PBS-EDTA eluate, and csbDNA from a trypsin eluate from the surface of blood cells were performed according to reference [5]. The extracellular DNA concentration was determined using an intercalating fluorescent dye PicoGreen [5]. The detection limits for DNA calculated to the initial blood volume were 0.4 ng/mL of blood in plasma, 2 ng/mL of blood in the PBS-EDTA eluate, and 20 ng/mL of blood in the trypsin eluate. The integral DNase activity in the blood plasma of healthy donors and patients was determined by an enzyme-linked immunosorbent assay (ELISA) as described in reference [6]. The ELISA sensitivity, defined as the minimal, statistically significantly determined activity of DNase 1 in a sample, was 0.004 U/mL of a sample. The variation coefficient at each point was not more than 4%.

Results were processed by the GraphPad Prism 5 software using the non-parametric Mann-Whitney test and the Spearman correlation coefficient.

## RESULTS AND DISCUSSION

Previously, we demonstrated that the blood of healthy donors, as well as that of patients with lung cancer [5] and stomach and colon cancers [7], contains constantly circulating DNAs that occur not only in blood plasma, but also in complexes bound to the surface of blood cells. A portion of csbDNA dissociates after the treatment of cells with PBS-EDTA buffer and is apparently bound to phospholipids and other anions of the cell membrane through bridges of divalent metal ions [8] or low-affinity interactions and is eluted with 9 buffer volumes (compared to the plasma volume); another csbDNA portion is removed from the cell surface by treating cells with a 0.125% trypsin solution and, apparently, is a part of the complexes with surface proteins of the blood cells [1].

**Fig. 1:**
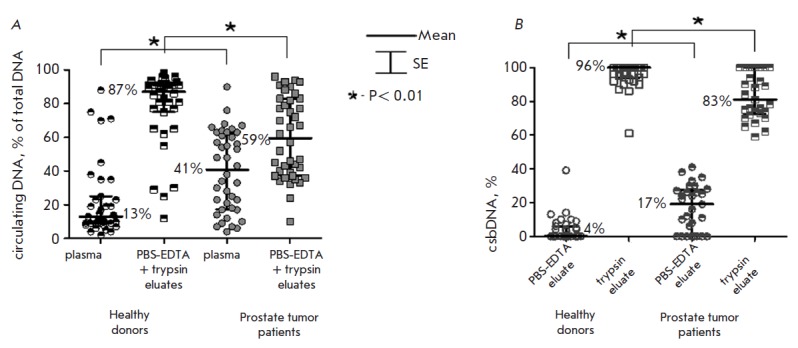
Relative amounts of cfDNA and csbDNA (*A*) and csbDNA (*B*) in the blood of healthy donors and PC patients

The present study investigates correlation relationships between the blood concentration of cfDNA and csbDNA in the norm and in prostate cancers (PCs). Since the cfDNA and csbDNA concentrations in patients with benign prostatic hyperplasia did not differ statistically significantly from their concentrations in the blood of prostate cancer patients (data are not shown), these groups of patients were combined into one group of patients with PCs. The ratio of cfDNA concentration to total circulating DNA (cf + csbDNA) concentration revealed a statistically significant (*P* < 0.01) increase in the fraction of cfDNA in PCs (40 ± 4%) compared to the norm (22 ± 4%) (*Fig. 1A*).

The PBS-EDTA eluate from the surface of the blood cells (i.e., weakly bound csbDNAs) of healthy donors and PC patients was found to contain 4 ± 1 and 17 ± 3% of the total amount of circulating DNAs bound to blood cells, respectively (*Fig. 1 B*), and these differences between healthy and sick males were statistically significant (*P * < 0.01).

The observed decrease in the csbDNA fraction and the simultaneous increase in the cfDNA fraction in the blood of PC patients may be due to the hydrolysis of csbDNAs by blood deoxyribonucleases. The data on DNase capability to hydrolyze nucleic acids bound to the cell surface are contradictory. Some authors believe that DNases can hydrolyze csbDNA [9], and according to others, DNases have little effect on the csbDNA concentration [8, 10].

**Fig. 2:**
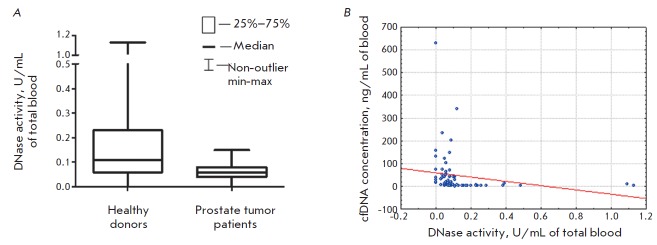
DNase activity in the blood plasma of healthy donors and PC patients (*A*); dependence of the cfDNA concentration on DNase activity in the blood plasma of healthy donors and PC patients (*B*)

The blood DNAse activity was determined using a previously developed enzyme-linked immunosorbent assay based on the hydrolysis of a DNA PCR fragment modified with fluorescein and biotin moieties [6]. An analysis of the cfDNA concentration and DNase activity in blood plasma from healthy donors and can cer patients revealed an appreciable (on the Chaddock scale) inverse correlation between these parameters (*R *= –0.57, *P* < 0.01) (*Fig. 2*).

These data demonstrate that DNases, despite a statistically significant (*P* < 0.01) decrease in their activity in the blood of PC patients compared to healthy donors (*Fig. 2*), can hydrolyze cfDNA and apparently are one of the factors negatively regulating the cfDNA concentration.

Investigation of the relationship between the blood plasma DNase activity and the csbDNA concentration demonstrated that the DNase activity is weakly correlated with the concentration of DNAs bound to the surface of blood cells via ionic interactions and is almost not correlated with the concentration of DNAs bound to cell surface proteins (*R *= –0.36, *P* < 0.01 and* R *= –0.28, *P* < 0.01, respectively). These data indicate that DNases do not actually hydrolyze csbDNAs strongly bound to cell surface proteins, do not contribute noticeably to the fragmentation process of csbDNA and its dissociation from the cell surface, and that the cfDNA concentration cannot increase due to the hydrolysis of csbDNA strongly bound to the cell surface.

**Fig. 3:**
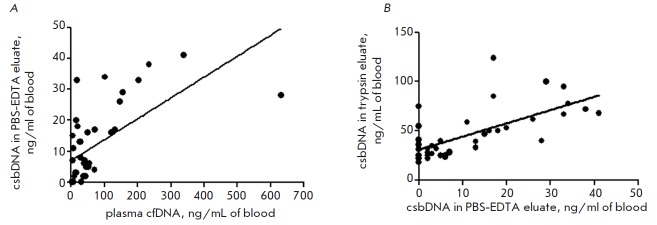
Relation between the plasma cfDNA concentration and the csbDNA concentration in PBS-EDTA eluate (*A*); relation between the PBS-EDTA eluate csbDNA concentration and the trypsin eluate csbDNA concentration (*B*)

The data from *in vitro *experiments [11] and the results of a study of blood csbDNA (*Fig. 1*) demonstrate that the main portion of csbDNA is removed from the cell surface upon treating cells with trypsin; i.e., it is bound to cell surface proteins. Based on the data on the correlation between the csbDNA concentration of the PBS-EDTA eluate and the DNase activity, it may be assumed that a portion of weakly bound csbDNAs can enter into an exchange with DNAs of the extracellular environment and blood plasma. An appreciable (on the Chaddock scale) direct correlation between the change in the plasma cfDNA concentration and the weakly bound csbDNA concentration (PBS-EDTA eluate) (*R *= 0.67, *P* < 0.01) in PC patients (*Fig. 3A*) confirms this supposition.

In addition, we found a direct correlation between the csbDNA concentration in the PBS-EDTA eluate and in the trypsin eluate in PC patients (*R *= 0.65, *P* < 0.001) (*Fig. 3B*), which may indicate a redistribution of DNA between these fractions of csbDNA.

Therefore, these data indicate a possibility of partial exchange between blood cfDNAs and csbDNAs. Indirectly, this fact is also confirmed by the data on the size of circulating DNA fragments. csbDNAs of the trypsin eluate are mainly represented by high-molecularweight (approximately 10–20 kb) DNA, and cfDNAs and csbDNAs of the PBS-EDTA eluate contain, apart from high-molecular-weight DNA, 200–500 bp fragments [3], which, apparently, can circulate as a part of one or another pool of circulating DNAs.

The causes of a decrease in the fraction of csbDNA in the total pool of circulating DNAs in the blood of PC patients are not known. It is likely that they are associated with a change in the structure of the cytoplasmic membranes of blood cells. Indeed, the development of oncologic diseases has been demonstrated to be accompanied by a change in the lipid ratio in blood cell membranes that leads to increased viscosity of the lipid bilayer, disruption of intermolecular protein-lipid interactions, and, as a consequence, disorganization of the protein composition, malfunction of the membrane cation transport systems, and disorganization of cell surface architectonics [12].

The pool of csbDNAs is known to be a valuable source of diagnostic material [4]. The possibility of cfDNA and csbDNA exchange suggests that, in the future, the most accurate diagnostic information could be obtained from an analysis of total blood-circulating DNA. Indeed, given that cfDNA and csbDNA can enter into an exchange (although the mechanisms of this process are not yet known), it is more accurate to use total blood-circulating (cfDNA + csbDNA) DNA to measure relative marker concentrations.

## Abbreviations

cfDNA: cell free DNA
csbDNA: cell-surface-bound circulating DNA
PC: prostate cancer
PBS-EDTA: 10 mM phosphate buffer (pH 7.5) with 0.15M NaCl and 5 mM EDTA
